# Glucagon Peptide Family and Adipose Tissue: Not that Intimate, just Acquaintances

**DOI:** 10.1210/endocr/bqaa006

**Published:** 2020-01-17

**Authors:** Diego Perez-Tilve

**Affiliations:** Department of Pharmacology and Systems Physiology, University of Cincinnati-College of Medicine, Cincinnati, Ohio

Emerging evidence demonstrates that gut-derived signals contribute to whole body metabolic control. The interest in searching for the mechanisms underlying such contributions has been particularly fostered over the past 15 years by the remarkable efficacy exhibited by different modalities of bariatric surgery on improving glucose homeostasis and lowering body weight, since those improvements correlate with dramatic changes in circulating levels of myriad gut-derived signals.

Among those signals there are peptides from the glucagon family, which includes glucagon itself, glucagon-like peptide 1 and 2 (GLP-1 and 2), and the related hormone gastric inhibitory peptide (GIP). In one way or another, these peptides play a key role in the control of glucose homeostasis and in the control of energy stores. As a matter of fact, mouse models with congenic global disruption of the receptors for those peptides (with the exception of GLP-2 receptor) exhibit a lean phenotype and resistance to diet-induced obesity. This has led to evaluation of the contribution these peptides make to the metabolic benefits of bariatric surgery. Furthermore, GLP-1 has become the foundation of a novel class of peptide-based therapeutics for the treatment of diabetes and obesity (1), which is now evolving toward multimimetic molecules containing different degrees of GLP-1, GIP, and/or glucagon activity (2).

At least when tested in animal models, these poly-agonists consistently offer superior efficacy improving glycemic control and reducing fat stores than single agonists. However, the reason for such increased efficacy is not well understood. It is likely related to the combined action of the agonists on their specific receptors, which are expressed in multiple, sometimes overlapping, tissues including some directly involved in the control of energy homeostasis.

Among those, the brain is clearly a predominant site of action. For example, expression of GLP-1 receptors in the brain’s critical feeding centers is well documented, and strong evidence indicates the role of GLP-1 action in those areas to influence both food intake and energy expenditure (1). The net balance of these activities results in fat mass loss. Recent evidence also supports a contribution of contribution of GIP receptor signaling in the brain to the control of energy stores (3,4). Whether brain glucagon signaling also contributes to the control of adiposity remains to be convincingly demonstrated.

In addition to the indirect effects of the glucagon family to influence fat mass as described above, the possibility that these peptides regulate fat stores by acting directly on either white or brown adipose tissue has also been hypothesized. This possibility has been considered nearly since the discovery of these peptides, supported by the fact that expression of their receptors can be detected (albeit at very low levels) and by observations on in vitro systems. These and other aspects suggesting a role for these peptides in regulating fat stores by directly acting on adipose tissue have been comprehensively examined in a recent review by Beaudry and Drucker (5). Supported by a thorough literature review, the authors systematically compare the bits of experimental evidence that have historically sustained the possibility of a direct control of adipocyte biology by the glucagon family of peptides with the latest results interrogating that question using state-of-the-art approaches, to which the authors have contributed in a very meaningful manner. This exercise leads to 2 paradoxical findings. The first is that evidence for receptor expression of any of these peptides has been documented, at least when considering different in vivo or in vitro models of different origin (ie, animal or human). The second is that the more refined the approach used to directly interrogate the role of such signaling systems in adipose tissue has become, the less prominent the contribution of such systems to the overall control of fat stores or energy balance appears to be. In other words, the receptors are there, but evidence for their functional role is lacking. This could be perhaps better summarized when comparing the effects of glucagon and GIP action. Pertaining to glucagon, its well-documented in vivo and ex vivo effects on lipid utilization, modulation of energy expenditure, and weight lowering benefits contrast with the relatively low levels of expression of its receptor in fat depots, including brown adipose tissue. Consequently, ablation of glucagon receptor expression exclusively from brown adipose tissue in mice (6) did not result in the reduced levels of adiposity exhibited by mice with global disruption of receptor expression. In the case of the GIP receptor, the actual contribution of GIP receptor signaling in adipocytes to the control of fat stores in vivo appears to be relatively modest, despite its well-documented expression in that tissue, although it has revealed potential contributions to other facets of metabolic control commonly associated with obesity (7).

Thus, a general corollary extracted from the review by Beaudry and Drucker (5) is that the glucagon family of peptides provide modest to no control of adiposity by acting directly on adipocytes. With the unfair advantage of hindsight, it could be argued that this outcome may seem evident, particularly when considering the low levels of receptor expression detected in adipose tissue. It could also be argued that a more definitive assessment of the actual contribution of direct action of this peptide on fat stores will require even more specific approaches such as temporally controlled ablation of receptor expression exclusively from adipose tissue. Ultimately, the degree of translatability to human physiology of those observations gathered in rodent models will always hold some degree of uncertainty due to experimental limitations.

In the interim, analysis of the role of the action of the glucagon peptide family on other cell types, such as the role of GIP receptor in immune cells, has recently revealed itself as an unexpected but critical site of action for the control fat stores (8). Hence, the mechanisms contributing to the control of energy balance by peptides from the glucagon family may not be hidden in plain sight after all.

## Additional Information

***Disclosure Summary:*** Dr. Perez-Tilve maintains research collaborations and receives funding from Novo Nordisk, Cohbar Inc., and MBX Biosciences Inc.
